# A Heterotypic Tridimensional Model to Study the Interaction of Macrophages and Glioblastoma In Vitro

**DOI:** 10.3390/ijms22105105

**Published:** 2021-05-12

**Authors:** María José Gattas, Ivana Gisele Estecho, María Amparo Lago Huvelle, Andrea Emilse Errasti, Eugenio Antonio Carrera Silva, Marina Simian

**Affiliations:** 1 Instituto de Nanosistemas, Universidad Nacional de San Martín, 25 de Mayo 1021, San Martín, Buenos Aires 1650, Argentina; majogattas@gmail.com (M.J.G.); mariamparo86@gmail.com (M.A.L.H.); 2 Instituto de Farmacología, Facultad de Medicina, Universidad de Buenos Aires, Paraguay 2155, Piso 9, Buenos Aires 1121, Argentina; ivanaestecho@gmail.com (I.G.E.); andreaerrasti@gmail.com (A.E.E.); 3 Instituto de Medicina Experimental (IMEX), Consejo Nacional de Investigaciones Científicas y Técnicas (CONICET), Academia Nacional de Medicina, Pacheco de Melo 3081, Buenos Aires 1425, Argentina

**Keywords:** glioblastoma multiforme, monocytes, macrophage polarization, 3D cultures, tumor-stromal interactions, CD206

## Abstract

Background: Glioblastoma multiforme (GBM) is the most frequent and aggressive primary brain tumor, and macrophages account for 30–40% of its composition. Most of these macrophages derive from bone marrow monocytes playing a crucial role in tumor progression. Unraveling the mechanisms of macrophages-GBM crosstalk in an appropriate model will contribute to the development of specific and more successful therapies. We investigated the interaction of U87MG human GBM cells with primary human CD14^+^ monocytes or the THP-1 cell line with the aim of establishing a physiologically relevant heterotypic culture model. Methods: primary monocytes and THP-1 cells were cultured in the presence of U87MG conditioned media or co-cultured together with previously formed GBM spheroids. Monocyte differentiation was determined by flow cytometry. Results: primary monocytes differentiate to M2 macrophages when incubated with U87MG conditioned media in 2-dimensional culture, as determined by the increased percentage of CD14^+^CD206^+^ and CD64^+^CD206^+^ populations in CD11b^+^ cells. Moreover, the mitochondrial protein p32/gC1qR is expressed in monocytes exposed to U87MG conditioned media. When primary CD14^+^ monocytes or THP-1 cells are added to previously formed GBM spheroids, both invade and establish within them. However, only primary monocytes differentiate and acquire a clear M2 phenotype characterized by the upregulation of CD206, CD163, and MERTK surface markers on the CD11b^+^CD14^+^ population and induce alterations in the sphericity of the cell cultures. Conclusion: our results present a new physiologically relevant model to study GBM/macrophage interactions in a human setting and suggest that both soluble GBM factors, as well as cell-contact dependent signals, are strong inducers of anti-inflammatory macrophages within the tumor niche.

## 1. Introduction

Glioblastoma multiforme (GBM), the highest grade, IV, glioma, is the most frequent and aggressive primary brain tumor. Current treatments are based on surgery followed by radio/chemotherapy. However, these therapies are not specific, and seldom successful in the long term; the median survival time for GBM patients is between 12.1 to 14.6 months [1]. The presence of other non-malignant cells within the tumor, comprised in large part by vascular and stromal cells together with inflammatory infiltrates such as microglia and macrophages [2], are increasingly being considered crucial in creating a tumor-promoting microenvironment.

Tissue resident macrophages of the central nervous system, also known as microglia, arise from the Yok sac lineage and can be maintained by self-renewing under homeostatic conditions. However, during the long lifespan of a human, peripherally derived macrophages may engraft into the brain under certain pathological conditions by enhancing monocyte trafficking [3,4]. More recently, it was suggested that infiltration of monocytes into the central nervous system occurs at a steady-state level under non-pathological conditions, and is implicated in brain plasticity [4]. Circulating monocytes, the precursors of macrophages, originate in the bone marrow and migrate across the vascular endothelium to peripheral tissues where they mature into macrophages and adopt different activation states [5]. In this context, infiltrating monocyte-derived macrophages are also found in the microenvironment of GBM, usually associated with perivascular necrotic areas [6,7]. According to the cytokines, chemokines, and growth factors present in the environment, macrophages polarize into different phenotypes. Although macrophage polarization is not a fixed state due to the plasticity and ability of these cells to integrate multiple signals, two major groups are classified as classical pro-inflammatory M1, and the regulatory and tissue repair M2 macrophages. Pro-inflammatory M1s are characterized by strong induction of pro-inflammatory cytokines, such as TNF-α, IL-6, IL-1β, and IL-12, high expression of CD64, and low levels of CD206 and CD163, and this subtype of macrophages are responsible for pathogen clearance. High levels of CD206, CD163, and MERTK expression characterize the anti-inflammatory M2 repertoire of macrophages that are involved in tissue repair and regulatory function [5,8,9].

In certain cancers, such as brain, lung, breast, and pancreatic, tumor progression is accompanied by an increase in macrophages with an immunosuppressive M2 phenotype. This phenomenon is specifically relevant in GBM where it has been reported that macrophages can represent up to 50% of the tumor mass [10,11]. Moreover, T cells are rare in GBM and consistently T-cell-based immunotherapies have failed to show improved patient response as compared to standard of care treatments. Thus, unraveling the mechanisms that lead to the positive interaction between brain tumor cells and macrophages will most certainly contribute to the development of specific and more successful therapies.

Here we investigated the interaction of human GBM cells (U87MG; [12]) with primary human monocytes or the immortalized monocyte-like cell line (THP-1; [13]) which were differentiated to macrophages in the presence of conditional medium or co-culture with the GBM cells cultured in 3-dimensions (3D). Our results show that only primary monocytes acquire an M2 immunosuppressive phenotype when incubated with U87 conditioned media in 2-dimensions (2D), as shown by the increased percentage of CD14^+^CD206^+^ and CD64^+^CD206^+^ cells in CD11b^+^ macrophages. Moreover, the mitochondrial protein p32/gC1qR, previously shown to be upregulated during tumor-associated macrophage polarization [14], is expressed in primary monocytes exposed to U87MG conditioned media. When primary monocytes or THP-1 cells were added to previously formed GBM spheroids, both are able to invade and establish within the spheroids. However, only primary monocytes differentiate and acquire a clear M2 phenotype characterized by the upregulation of CD206, CD163, and MERTK surface markers on the CD11b^+^CD14^+^ population after 7 days of co-culture. These data suggest that there are GBM soluble and, additionally, distinct cell-contact dependent signals that induce anti-inflammatory macrophages within the tumor niche. Our results present a new physiologically relevant model to study GBM/macrophage interactions in a human setting. This type of cell culture model may contribute to the development of strategies that target macrophages and lead to successful GBM therapies. We propose this approach could be an attractive option for testing drugs in a more suitable microenvironment and with a physiologically relevant macrophage population.

## 2. Results

### 2.1. Culture of Primary Monocytes with Conditioned Media of U87MG Cells Leads to an Increased Expression of M2 Phenotypic Markers

**Figure 1:**
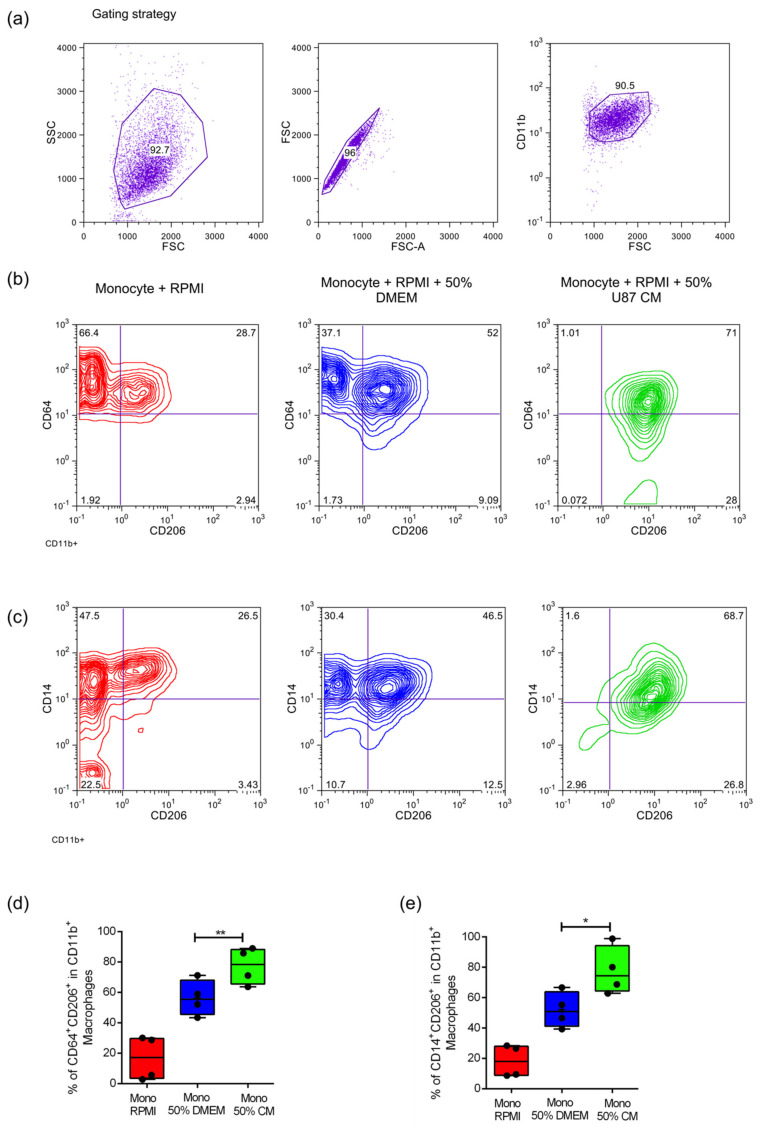
U87MG conditioned media induces M2 polarization of primary human monocytes. Sorted primary human CD14^+^ monocytes (Mo) were incubated for 5–7 days in complete RPMI (RPMI), complete RPMI + 50% complete DMEM low glucose (50% DMEM) and complete RPMI + 50% U87MG conditioned media (CM) (50% CM) to obtain monocyte-derived macrophages as explained in materials and methods. (**a**) Gating strategy to discriminate CD11b^+^ macrophages at day 7. (**b**,**c**) Representative contour plots showing the percentage of CD64^+^ CD206^+^ and CD14^+^ CD206^+^ in CD11b^+^ cells after 7 days of incubation with RPMI, 50% DMEM and 50% CM. (**d**,**e**) Independent data showing the increased percentage of CD64^+^ CD206^+^ and CD14^+^ CD206^+^ respectively, of CD11b macrophages under CM treatment (*n* = 4). In both cases two tailed paired *t*-tests were performed *: *p* ≤ 0.05; **: *p* ≤ 0.01.

In order to establish the impact of GBM-produced soluble factors on primary human monocytes as well as on the immortalized monocyte-like THP-1 human cell line, conditioned media was collected from U87MG cells as described in materials and methods. The media was centrifuged to eliminate any remaining cell debris, and frozen at −20 °C until used. In all cases, the conditioned media was diluted with 50% fresh media before adding it to the monocytes. Human CD14^+^ monocytes were obtained by positive selection from peripheral blood mononuclear cells (PBMCs) as described previously [7,15] and cultured in the presence or absence of conditioned media during 5–7 days. After harvesting, polarization markers were analyzed using CD11b, CD14, CD64, CD206, CD163, MERTK, and viability dye. Our results show that treatment with conditioned media increased the percentage of CD11b^+^ macrophages expressing CD64^+^CD206^+^ as well as CD14^+^CD206^+^ double-positive cells (Figure 1a–d) which indicate a skewing towards an anti-inflammatory M2 phenotype. No significant differences were observed in CD163 and MERTK expression (data not shown). We did not observe significant changes in M2 polarization markers in the THP-1 cell line in the presence of U87MG conditioned media on its own or after activation with phorbol 12-myristate 13-acetate (PMA) (Supplementary Figure S1a,b).

**Figure 2:**
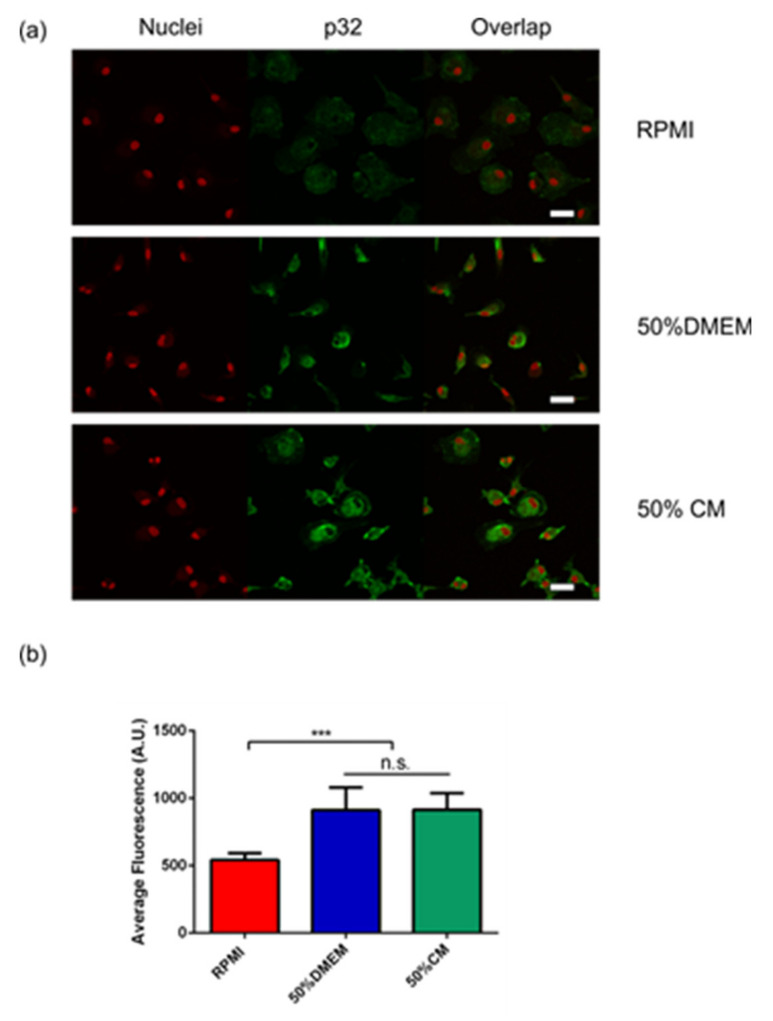
Primary human monocytes express p32/gC1qR when M2 markers increase. (**a**) Primary human monocytes were incubated with complete RPMI (RPMI), RPMI + 50% complete DMEM low glucose (50%DMEM) and RPMI + 50% U87MG conditioned media (50% CM) for 5 days and were subsequently fixed and stained for p32/gC1qR as explained in materials and methods. (**a**) Representative images of stained cells; nuclei are counterstained in red (red dots) and p32/gC1qR is shown in green (Alexa 488). (**b**) Statistical analysis showing the relative fluorescence in arbitrary units (A.U.) for cells incubated in the conditions explained above. A statistically significant increase in p32/gC1qR staining was detected in cells incubated in 50% DMEM and in 50% CM, *n* = 3. A One-way Anova was performed ***: *p* ≤ 0.001. Scale bar: 20 μm.

Additionally, p32/gC1qR, a mitochondrial protein expressed on the cell surface of tumor-associated macrophages [14] was also increased in the monocytes incubated with 50% DMEM and when conditioned media was added, in agreement with the described increase in M2 markers (Figure 2a,b).

Finally, THP-1 cells did not show changes in the expression of p32/gC1qR as measured by immunofluorescence (Supplementary Figure S1c) supporting the notion that this cell line is not modulated by the experimental conditions established in this paper.

### 2.2. Primary Human Monocytes Infiltrate Human GBM Spheroids and Alter Their Growth and Sphericity

**Figure 3:**
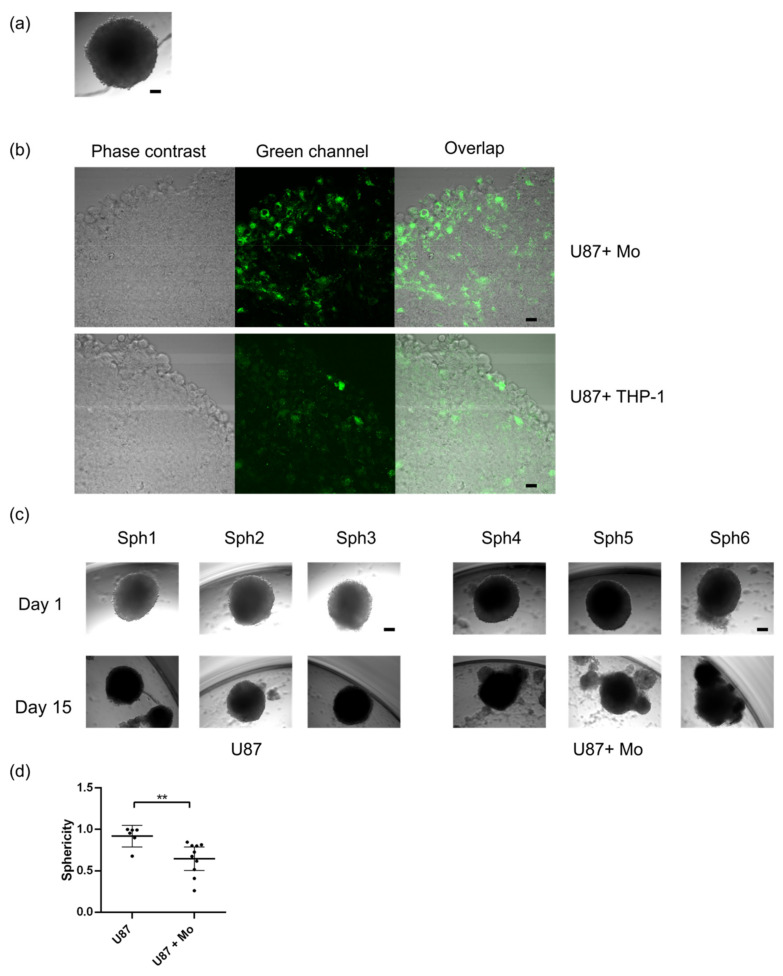
Impact of primary human monocytes on the growth of U87MG spheroids. (**a**) The appearance of 7 day-hanging drop spheroid generated from U87MG cells. (**b**) Phase contrast and confocal images of spheroids infiltrated with primary human monocytes (Mo) and THP-1 cells (in green) after 5 days of co-culture. (**c**) Representative images of U87MG spheroids at days 1 and 15 after infiltration with primary human monocytes showing loss of sphericity in the co-culture conditions. (**d**) Independent data showing loss of sphericity in spheroids without (*n* = 6) and with (*n* = 9) primary human monocytes. A two-tailed unpaired *t*-test was performed **: *p* ≤ 0.01. Scale bar: 50 μm.

Having established that U87MG conditioned media led to the polarization of the primary human monocytes, we next investigated if they would be capable of infiltrating pre-formed GBM spheroids. To do so we set up a hanging-drop method and determined that cultures originating from 3000 U87MG cells formed reproducible GBM spheroids. Spheroids were allowed to develop for 7 days, the time at which they were formed by 200–300,000 cells each. Figure 3a shows a representative image of a 7-day spheroid when it was dropped into a U-shaped well. The day after, 40,000 primary monocytes or THP-1 cells were added to the cultures. To visualize their destination within the cultures we pre-stained them with a live-cell fluorescent dye. Figure 3b shows confocal images of sections of U87MG spheroids infiltrated with primary human monocytes and THP-1 cells respectively. Next, we evaluated the impact of the monocytes on the growth of the U87MG spheroids. Figure 3c shows representative images of spheroids infiltrated with CD14+ monocytes after 15 days in culture. Infiltrated primary immune cells did not have a significant impact on the volume of the spheroids (not shown), however, when sphericity was analyzed we determined that the presence of monocytes induced a loss of sphericity in the U87MG cultures (Figure 3d), a characteristic previously associated to malignancy [16,17]. Interestingly, when the spheroids were infiltrated with THP-1 cells no changes were registered in their size or shape (Supplementary Figure S2). Thus, our results imply that the infiltration of immune cells into spheroids does not necessarily lead to alterations in their growth rate or pattern. Moreover, we determined that the response to the spheroids depends on the cell type used; in particular, only primary human monocytes altered the sphericity of the 3D cultures.

### 2.3. Infiltrated Monocytes Acquire an M2-Phenotype When They Invade the GBM Spheroids

**Figure 4:**
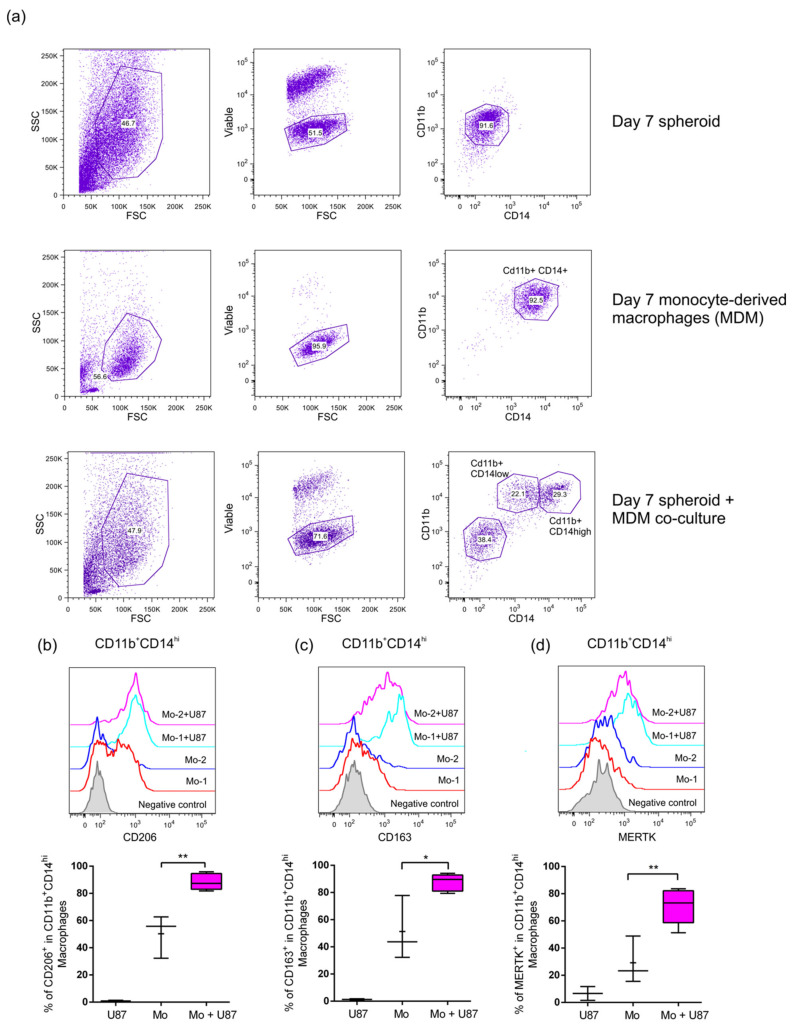
CD14^+^ monocytes infiltrating U87MG spheroid are skewed to a strong M2 phenotype. U87MG spheroids were co-cultured with sorted CD14^+^ human monocytes (Mo) for 7 days. Cells were dissociated and the phenotype of the infiltered monocyte-derived macrophages (MDM) was determined by flow cytometry. U87MG spheroids alone and 7-day MDM cultures were used as controls. (**a**) Gating strategies to discriminate CD11b^+^ CD14^hi^ macrophages from the heterotypic co-cultures and control culture conditions are shown. (**b**–**d**) Representative plots as well as the independent data of each condition showing the percentage of CD206^+^ (**b**), CD163^+^ (**c**), and MERTK^+^ (**d**) in the CD11b^+^ CD14^hi^ gating. Two-tailed unpaired *t*-tests were performed *: *p* ≤ 0.05, **: *p* ≤ 0.01. *n* = 5.

**Figure 5:**
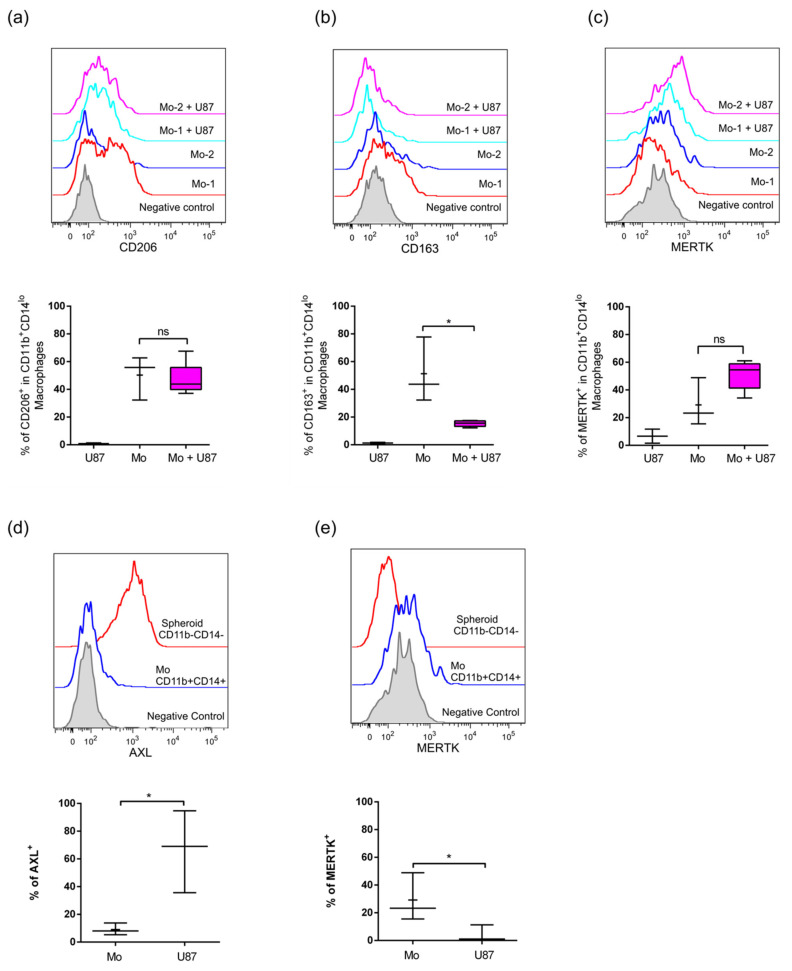
Heterotypic co-cultures show the presence of unskewed CD11b^+^CD14^low^ macrophages and high expression of AXL in U87MG cells. Using the gating strategy described in Figure 4a–c, M2 polarization markers were analyzed in the CD11b^+^ CD14^low^ subpopulation and compared to 7-day monocyte-derived macrophages. Representative histograms as well as the independent data showing the expression of CD206^+^ (**a**), CD163^+^ (**b**) and MERTK^+^ (**c**) in the CD11b^+^ CD14^low^ gating. Percentage of AXL^+^ (**d**) and MERTK^+^ (**e**) cells in U87MG as compared to monocyte-derived macrophages. Two-tailed unpaired *t*-tests were performed, *: *p* ≤ 0.05, (*n* = 3).

Tumor infiltrated leukocytes play a critical role in tumor establishment, development, and expansion. In this sense, understating cell communication and instruction among them is fundamental for tumor outcome. Instruction can come through soluble factors; however, cell-cell contact is one of the strongest reprogramming signaling cues of the tumor microenvironment. Having shown that human monocytes and THP-1 cells can infiltrate GBM U87MG spheroids and that only primary monocytes impacted their growth pattern, we next evaluated what types of monocyte-derived macrophages (MDM) were promoted in this microenvironment. Co-cultures were allowed to develop for 7 days as explained above and were subsequently processed to analyze the phenotype of the infiltrate as explained in materials and methods. To distinguish the MDM from the GBM cells we used CD11b and CD14 markers, which allow us to differentiate two sub-populations based on the acquisition of high and low levels of CD14 expression (CD11b^+^CD14^high^ and CD11b^+^CD14^low^) (Figure 4a). The strongest M2 polarization was observed in CD11b^+^CD14^high^ macrophages which induced high levels of CD206, CD163, and MERTK (Figure 4b–d). This clear shift to anti-inflammatory and regulatory phenotype is concordant with macrophages phenotype isolated from GBM tissue samples [7]. Interestingly, when we analyzed the phenotype of macrophages from the CD11b^+^CD14^low^ sub-population we did not observe markers associated with M2 skewing (Figure 5a–c), which could be indicating a second not totally polarized population or a second totally different sub-population. This data could also indicate that macrophages developed during the interaction with U87MG GBM are heterogeneous. Noteworthy, when we compared the tyrosine kinase receptors AXL and MERTK in U87MG cells vs. MDM, AXL was highly expressed only in glioma cells, whilst MERTK was expressed in MDM (Figure 5d,e) which denote the difference in the cell type source during heterotypic co-culture. Finally, THP-1 cells did not show significant changes in their polarization profile (Supplementary Figure S3) when co-cultured within the U87MG spheroid, in accordance with their lack of impact on the 3D culture sphericity. Thus, the 3D-heterotypic model established with primary human monocytes in combination with U87MG cells recapitulates human GBM to a greater extent than the cultures utilizing THP-1 cells.

## 3. Discussion

The aim of the current paper was to establish a human 3D-heterotypic culture system where the interactions of GBM cells and infiltrating macrophages were recapitulated in a physiologically relevant manner. There is an increased interest in establishing improved biological systems that better represent the in vivo physiological conditions. In this sense, a lot of energy is currently being directed towards developing 3D cultures as well as organoid cultures [18]. In this study, we used the widely characterized U87MG human GBM cell line and CD14^+^ human primary monocytes, or the THP-1 human leukemia monocytic cell line to evaluate the impact of soluble factors as well as cell-to-cell dependent signaling on the behavior of both the GBM cells and the monocytes. Our results show that U87MG-derived soluble factors skew circulating monocytes to differentiate into CD11b^+^ macrophages expressing CD64^+^CD206^+^ as well as CD14^+^CD206^+^ double-positive cells. Tumor cells attract and reprogram innate immune cells including tumor-associated macrophages to support tumor growth and metastatic spread. Interestingly, many cytokines, growth factors, or different ligands of macrophage receptors are expressed and secreted by tumor cells to coax macrophages into supporting vascularization and tumor growth [19,20]. Even though soluble factors are able to target infiltrating immune cells, the most effective way to reprogram infiltrating monocytes to tumor-promoting macrophages is by cell contact. In concordance, we set a heterotypic 3D-cultures system using U87MG spheroids together with CD14+ human primary monocytes, or THP-1 cells. In both cases, the immune cells invaded the spheroids and established within them. However, only primary monocytes were able to induce significant changes in sphericity, an indicator of invasion and metastasis [16,17]. Furthermore, primary monocytes showed a strong M2 polarization inside the spheroids, particularly when gating on CD11b^+^CD14^high^ macrophages which showed high levels of CD206, CD163, and MERTK. We also found a second macrophage subpopulation characterized by CD11b^+^ and low expression of CD14, which did not show significant changes in the expression of M2 markers. This result could be indicating that the 3D culture contains macrophages at different polarization stages or, directly, two different types of macrophages developed at the same time. Macrophages are highly dynamic cells whose molecular profiles are substantially influenced by specific environmental cues and a mixture of macrophage types were previously found in GBM human samples as well as in animal models [21], suggesting that we could have at least two different subpopulations of macrophages. Further studies are required to answer this question. No significant changes in M2 polarization markers were detected when we used THP-1 cells. Thus, even though both cell lines are able to invade the GBM 3D cultures, only primary human monocytes establish a physiologically relevant phenotype that recapitulates the macrophage phenotype identified in human GBM tissue samples.

Therapies targeting the immune system are revolutionizing cancer treatment. In the case of GBM, the predominance of tumor-associated macrophages is associated with the limited success of these strategies. Most current immunotherapies take advantage of the adaptive anti-tumor immunity and include immune checkpoint blockade therapy, vaccination therapy, CAR-T therapy, and oncolytic virus therapy. However, 30–40% of the cell composition of gliomas is accounted by the presence of macrophages, being 85% of them derived from bone marrow infiltrating monocytes and macrophages [22] and the remaining 15% corresponds to locally resident microglia [23]. Thus, the establishment of adequate models that precisely recapitulate the interaction of tumor and immune cells are critical to study and test novel therapeutic strategies. In this sense, currently, there are only a handful of reports, to our knowledge, showing diverse strategies to develop relevant pre-clinical culture systems where tumor cells are combined with macrophages [24]. Henrich et al. [25] recently developed a 3D-bioprinted murine GBM model. They used mouse GL261 GBM cells [26] together with the RAW264.7 mouse macrophage cell line [27]. The authors show that when co-cultured in bioprinted minibrains, RAW264.7 cells are skewed towards an M2 phenotype as determined by the upregulation at the transcriptional level of CD206, and arginase-1. Moreover, the macrophages induce GBM cell invasion within the mini-brains. Leite et al. [28], on the other hand, utilized human GBM cell lines (U87MG, SNB-19 [29] and UP-007 [established in their laboratory]) together with an immortalized human fetal microglia cell line, CHME3 [30]. The co-cultures were established by seeding the two cell types directly in hyaluronic acid-based hydrogels, in different ratios. The authors report that the presence of the microglia promoted the proliferation and invasion of the GBM cells, as well as drug resistance. However, even though the authors show that the CHM3 cells are responsive to environmental stimuli such as LPS and IFNγ, they did not analyze the phenotype of the microglia after several days in co-culture. In this paper, we decided to use spheroids generated by the hanging-drop method, as it is a simple and reproducible strategy. Our initial experiments determined that both primary human monocytes and THP-1 cells have the intrinsic capacity to invade the spheres and remain viable within them. However, primary monocytes turned out to be highly responsive to both soluble factors and signals induced by the direct contact established with the GBM cells. Interestingly, the direct cell-cell contact established in the 3D-cultures was the strongest inducer of macrophages polarization, with high expression levels of CD206, CD163, and MERTK. In this regard, CD206 and MERTK expression were found within the immune infiltrate in multiple solid tumors, highlighting its potential role in cancer immunity [20]. Further experiments to shed light on the functional crosstalk between neoplastic cells and the tumor microenvironment will enable to dissect the complex cell-cell interactions and improve the current therapeutic strategies.

It is still not well understood whether macrophage heterogeneity is a result of their reprogramming status in response to the tissue environment or due to spatial-temporal localization inside the tumor. In this sense, single-cell RNA-seq analyses of GBM biopsies demonstrated that infiltrating macrophages frequently co-express canonical pro-inflammatory (M1) and alternatively activated (M2) genes. Similar studies provided insights about the spatial localization of a macrophage core signature highly present within the tumor core, while cells from the periphery expressed an evident microglia signature [31].

Our results clearly show that microenvironmental cues are not only the result of soluble factors produced by the tumor cells but that the physical interaction between the two cell types creates a more complex scenario that is probably the results of a bi-directional paracrine cross-talk. Future experiments will allow us to determine whether direct cell-cell contact is necessary to induce monocyte differentiation, or if reciprocal soluble signals between both cell types mediate the observed changes. In conclusion, we provide an innovative and simple 3D-culture model to study monocyte/GBM cell interactions that should, additionally, be valuable as a testing platform for novel therapeutic strategies.

## 4. Materials and Methods

### 4.1. Cell Culture

U87MG cells were kindly provided by Dr. Carolina Perez Castro from IBioBA, Buenos Aires, Argentina, and cultured in low glucose Dulbecco’s Modified Eagle Medium (DMEM), 4 mM L-Glutamine, 110 mg/L sodium lithium, and 10% FBS (complete DMEM low glucose medium). THP-1 cells were kindly provided by Dr. Federico Coluccio-Leskow from the School of Exact and Natural Sciences, University of Buenos Aires, and cultured in RPMI 1640 with 4 mM L-glutamine and 10% FBS. 

### 4.2. Blood Sample and Purification of Human CD14+ Monocytes

Peripheral blood mononuclear cells (PBMCs) were obtained from healthy volunteer donors who had given written consent and has not taken any non-steroidal anti-inflammatory drugs for 10 days prior to sampling, as previously described [5,8,32]. Briefly, PBMCs from healthy donors were isolated by Ficoll-Hypaque (GE, Chicago, IL, USA) density gradient centrifugation, and a positive selection of CD14+ monocytes was performed using an EasySep Human CD14 Positive Selection Kit (StemCell Tech, Vancouver, BC, Canada) as indicated by the manufacturer. The study was conducted according to the guidelines of the Declaration of Helsinki and the protocol was approved by the Institutional Ethics Committee of the National Academy of Medicine, Argentina.

### 4.3. Collection of Conditioned Medium

U87MG cells (3 × 10^5^) were seeded in 24-well-flat-bottom-plates and grown for 48 h in a complete DMEM low glucose medium. Cell medium was collected and centrifuged for 5 min at 300× *g* to remove cell debris and frozen at −20 °C until used.

### 4.4. Stimulation of CD14+ Cells and THP1 with Conditioned Medium

Sorted CD14+ monocytes or THP-1 cell line (2 × 10^5^) were seeded in 24-well-flat-bottom-plates containing complete RMPI medium (supplemented with 10% FBS and 1% penicillin-streptomycin (PS)) or a mixture containing 50% of complete RPMI plus 50% of the conditioned medium; as well as 50–50% of complete RPMI with low glucose DMEM. Cells were grown for 5 or 7 days in the incubator (Thermo) at 37 °C, and 5% CO_2_.

### 4.5. p32/gC1qR Staining

Cells were fixed in PFA 4% for 20 min at RT and then blocked in BSA 3%. Primary antibodies against p32 (a kind gift of Dr. Tambet Teesalu) were incubated at a 1:500 dilution overnight at 4 °C. The secondary antibody, anti-rabbit Alexa 488 (Abcam, Cambridge, UK) was incubated at RT for 1 h. RedDot (Biotium, Freemont, CA, USA) was used for nuclear staining. Nonspecific binding was addressed by incubating with secondary antibodies alone. Images were taken by confocal microscopy (Olympus FV-1000, Tokyo, Japan) and analyzed with Image J.

### 4.6. Tumor Spheroid Generation

To generate cell spheroids, we adapted the previously published hanging drop method [33]. Briefly, 3 × 10^4^ U87MG cells were seeded on the cover of 48-well plates in 20 μL drops. Covers were then inverted and incubated for 72 h until spheroids were fully formed. They were then transferred into individual wells containing 100 μL of U87MG medium. Spheroids were fed every 48 h carefully aspirating 50 μL of medium and replacing it with the same volume of fresh medium.

### 4.7. Monocyte-Spheroid Co-Culture

Once U87MG spheroids were grown for 7 days, 4 × 10^4^ CD14+ monocytes were added per spheroid. This co-culture was incubated for 15 days and changes were recorded every two days approximately. Spheroids with 5 and 7 days of growth were used for flow cytometry measurements while the others were used to measure growth and sphericity. The same procedure was carried out using THP-1 cells. In order to visualize macrophage infiltration within spheroids; THP1 and monocytes were previously dyed with Biotium Cellbrite green (CellBrite^®^ Cytoplasmic Membrane Dyes, Freemont, CA, USA) for 2 h at 37 °C at a 1/200 dilution in RPMI. After staining, cells were centrifuged at 3000× *g* for 5 min and resuspended in RPMI, and added to the sphere cultures. Finally, infiltrated spheroids were fixed for confocal microscopy. All images were analyzed with ImageJ software.

### 4.8. Spheroid Dissociation

Spheroids were collected and then dissociated by vigorous pipetting in PBS-FBS2%-EDTA-1mM buffer. Next, the cells were passed through 50 μm filters to generate a suspension of individual cells. These cells are then collected, centrifuged for 5 min 300× *g*, and stained for flow cytometry.

### 4.9. Surface and Intracellular Staining and Flow Cytometry Analysis

The surface staining for the phenotypic characterization of macrophages was performed using APC/Cy7-CD11b (Cat#101225, RRID:AB_830641), APC-CD64 (Cat#305013, AB_1595539), PercP/Cy5.5-CD163 (Cat#333625, RRID:AB_2,650629), AF488-CD206 (Cat#321113, RRID:AB_571874) and PECy7-CD14 (Cat#325618, AB_830691) (Biolegend, San Diego, CA, USA) following standard protocols. Briefly, the harvested cells were washed with PBS and blocked in PBS/2% FBS on ice for 30 min. The cells were washed with PBS and the respective antibody cocktails (prepared in PBS/2% FBS) were added to the cell pellet and incubated for 30 min on ice. A fixable viability dye was used according to the manufacturer’s instructions to gate on live cells. After washing, the cells were fixed with a Cytofix/Cytoperm Kit (BD Bioscience Cat#554715, San José, CA, USA), washed again, and analyzed in a FACS Canto I (Becton Dickinson, San José, CA, USA). All analysis was carried out with FlowJo software (Tree Star). AXL and MERTK expression were evaluated after fixation and permeabilization (Cytofix/Cytoperm Kit, BD San Jose, CA, USA) using biotin-conjugated goat anti-human AXL (R&D Systems Cat#AF154, RRID:AB_354852; Minneapolis, MN, USA) and mouse anti-human APC-MERTK (R&D Systems Cat#FAB8912A, RRID:AB_357213, Minneapolis, MN, USA). PE-conjugated Streptavidin (BioLegend Cat#405203, San Diego, CA, USA), was used for AXL signal detection [8].

## Data Availability

The data presented in this study is available on request from the corresponding author. The data are not publicly available due to privacy.

## Supplementary Materials

The following are available online at https://www.mdpi.com/article/10.3390/ijms22105105/s1. Figure S1: U87MG conditioned media doesn’t enhance macrophage polarization in THP-1 cells. THP-1 cells were incubated for 7 days in complete RPMI, complete RPMI with PMA (10 ng/mL), complete RPMI + 50% U87MG Conditioned Media (CM) and complete RPMI with PMA (10 ng/mL) + 50% U87MG Conditioned Media (CM) to see macrophages differentiation and polarization. No significant differences are shown in the expression of CD64 in CD11b+ gated cells (Figure a, second panel) neither in the expression of CD206 (a, third panel) or in the expression of CD163 (b) (*n* = 3). THP-1 cell line expresses p32/gC1qR in three different treatments. Cells were incubated with RPMI, RPMI + 50% complete DMEM low glucose (50%DMEM) and RPMI + 50% U87MG conditioned media (50% CM) for 5 days and were subsequently fixed and stained for p32 as explained in materials and methods. Representative images of stained cells; nuclei are counterstained in red (red dot) and p32 is shown in green (Alexa 488) (c) no significant difference are shown in the three treatments. Figure S2: THP-1 cells infiltrating U87MG spheroid are not skewed to a M2 phenotype. We used gated CD64 + CD11b + THP-1 cells inside the spheroid or cultured in complete fresh RPMI (control) to analyze further macrophage differentiation (a). Representative histograms show no differences in the expression of CD206+, CD163+ or MERTK+ between this THP-1 in co-culture or the control (b). Figure S3: Impact of THP-1 cells on the growth of U87MG spheroids. (a) Representative images of U87MG spheroids at days 1 and 15 after infiltration with THP-1 cells. (b) Independent data showing no differences in the sphericity of spheroids without (*n* = 10) and with (*n* = 10) THP-1 cells. A two-tailed unpaired t-test was performed **: *p* ≤ 0.01. Scale bar: 50 m.
