# Supplementary material for: A Heterotypic Tridimensional Model to Study the Interaction of Macrophages and Glioblastoma In Vitro
Source: Int J Mol Sci. 2021 May 12;22(10):5105. doi: 10.3390/ijms22105105 (PMC8151206; doi:10.3390/ijms22105105)
Supplement: Supplementary file 1 [file ijms-22-05105-s001.zip › ijms-1179762-supplementary.pdf]

Supplementary Figures

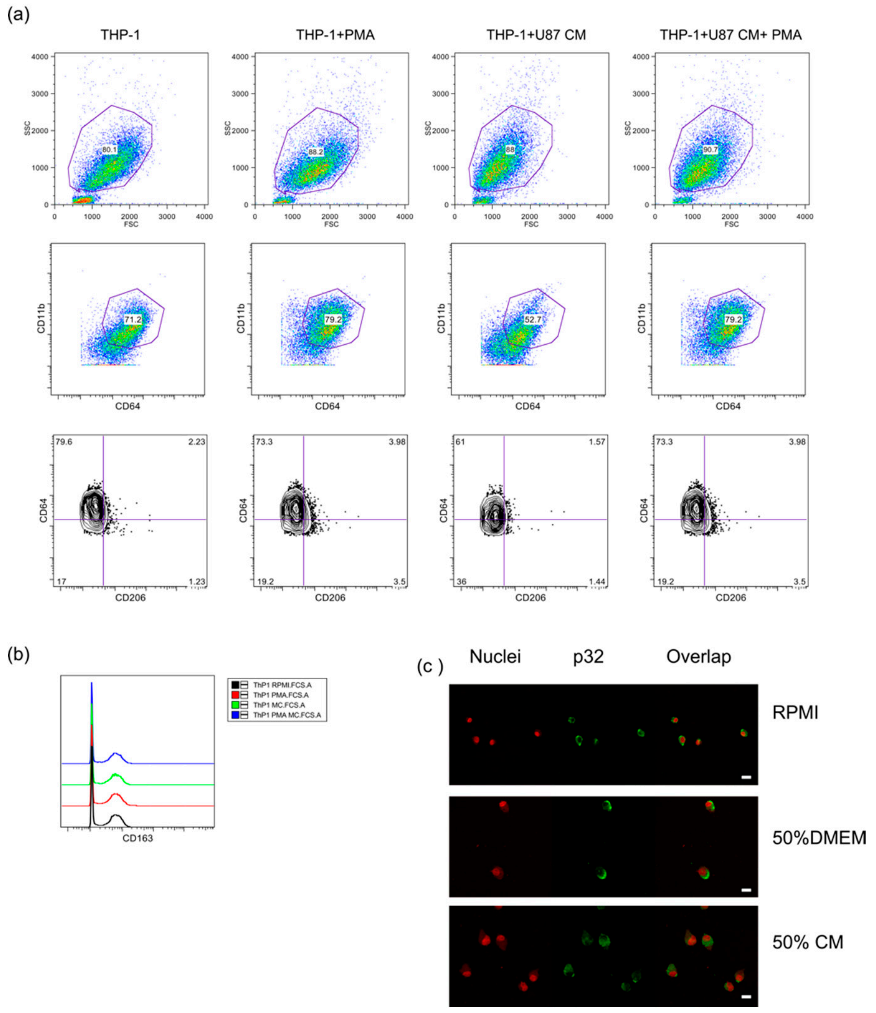

Supplementary Figure S1: U87MG conditioned media doesn´t enhance macrophage polarization in THP-1 cells. THP-1 cells were incubated for 7 days in complete RPMI, complete RPMI with PMA (10ng/ml), complete RPMI + 50% U87MG Conditioned Media (CM) and complete RPMI with PMA (10ng/ml) + 50% U87MG Conditioned Media (CM) to see macrophages differentiation and polarization. No significant differences are shown in the expression of CD64 in CD11b+ gated cells (Figure a, second panel) neither in the expression of CD206 (a, third panel) or in the expression of CD163 (b) (N=3). THP-1 cell line expresses p32/gC1qR in three different treatments. Cells were incubated with RPMI, RPMI + 50% complete DMEM low glucose (50%DMEM) and RPMI + 50% U87MG conditioned media (50%CM) for 5 days and were subsequently fixed and stained for p32 as explained in materials and methods. Representative images of stained cells; nuclei are counterstained in red (red dot) and p32 is shown in green (Alexa 488) (c) no significant difference are shown in the three treatments.

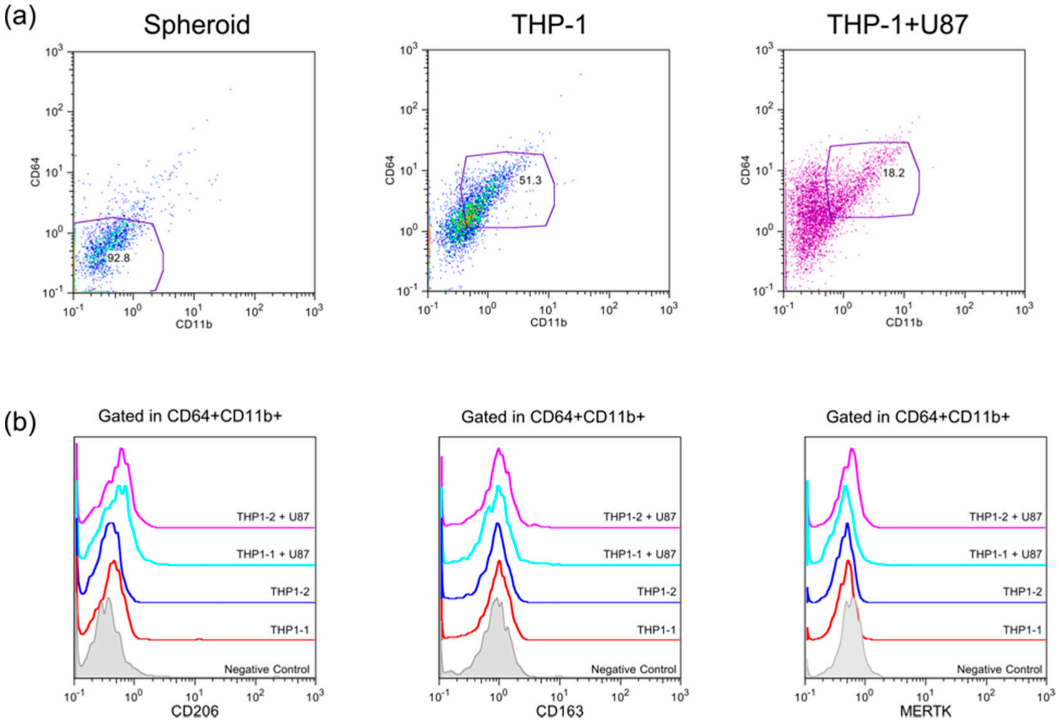

Supplementary Figure S2: THP-1 cells infiltrating U87MG spheroid are not skewed to a M2 phenotype. We used gated CD64+CD11b+ THP-1 cells inside the spheroid or cultured in complete fresh RPMI (control) to analyze further macrophage differentiation (a). Representative histograms show no differences in the expression of CD206+, CD163+ or MERTK+ between this THP-1 in co-culture or the control (b).

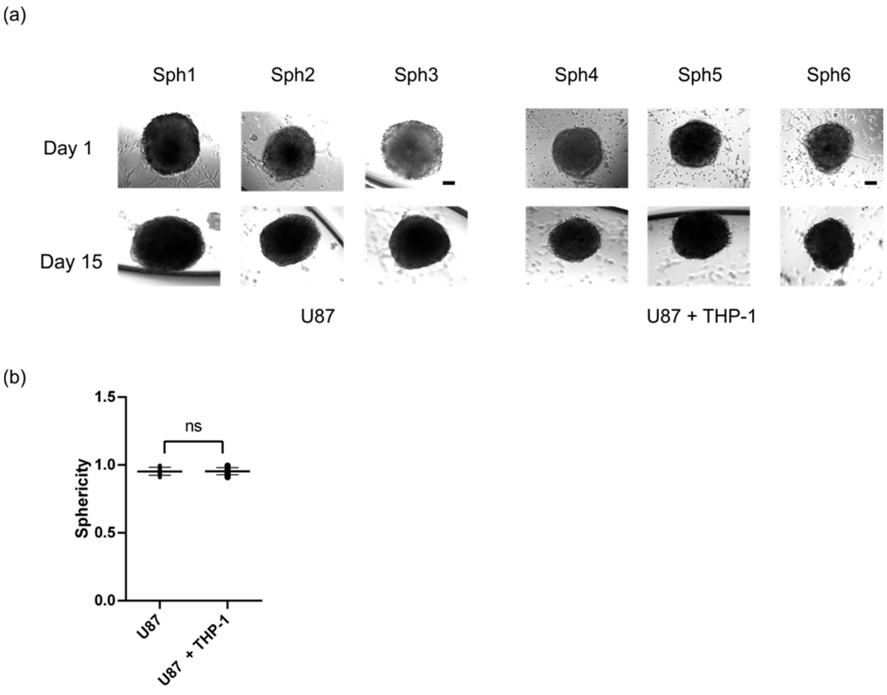

Supplementary Figure S3: Impact of THP-1 cells on the growth of U87MG spheroids. (a) Representative images of U87MG spheroids at days 1 and 15 after infiltration with THP-1 cells. (b) Independent data showing no differences in the sphericity of spheroids without (N=10) and with (N=10) THP-1 cells. A two-tailed unpaired t-test was performed **: $p \leq .01$. Scale bar: 50 µm.
